# Nanomaterials design for super-degenerate electronic state beyond the limit of geometrical symmetry

**DOI:** 10.1038/s41467-018-06244-8

**Published:** 2018-09-14

**Authors:** Naoki Haruta, Takamasa Tsukamoto, Akiyoshi Kuzume, Tetsuya Kambe, Kimihisa Yamamoto

**Affiliations:** 1 Institute of Innovative Research, Tokyo Institute of Technology, Yokohama, 226-8503 Japan; 2 ERATO, JST, Kawaguchi, Saitama 332-0012 Japan

## Abstract

Spherical atoms have the highest geometrical symmetry. Due to this symmetry, atomic orbitals are highly degenerate, leading to closed-shell stability and magnetism. No substances with greater degrees of degeneracy are known, due to geometrical limitations. We now propose that realistic magnesium, zinc, and cadmium clusters having a specific tetrahedral framework possess anomalous higher-fold degeneracies than spherical symmetry. Combining density functional theory calculations with simple tight-binding models, we demonstrate that these degeneracies can be attributed to dynamical symmetry. The degeneracy condition is fully identified as an elegant mathematical sequence involving interatomic parameters. The introduction of dynamical symmetry will lead to the discovery of a novel category of substances with super-degenerate orbitals.

## Abstract (web summary)

No substances with greater degrees of degeneracy than spherical atoms are known, due to geometrical limitations. In this work the authors combine density functional theory and tight-binding models to predict metal clusters with higher-fold degeneracies than spherical atoms, which are ascribed to dynamical symmetry.

## Introduction

Symmetry is the most fundamental concept in both physics and chemistry^1–3^, and the basic properties of classical and quantum systems can be derived based on this concept. In real space, atoms have the highest geometrical symmetry, three-dimensional spherical symmetry *O*(3). As a result of this symmetry, these species possess atomic orbitals with the high degrees of degeneracy, such as *d* orbitals, which have a fivefold degeneracy. This degeneracy in turn leads to certain properties, such as closed-shell stability and magnetism. However, species having higher degrees of degeneracy than atoms have not yet been known, due to the limitations of geometrical symmetry. It is of interest to consider whether or not it is possible to overcome this limitation.

Recently, the concept of a superatom has been proposed and developed^4–8^. A superatom is analogous to an atom but with a higher-order structure: highly symmetrical metal clusters possess delocalized molecular orbitals, the shapes of which are just like those of atomic orbitals. This analogy can be understood based on the three-dimensional spherical jellium model^9^. As the full occupation of superatomic orbitals results in stable closed shells, the numbers of valence electrons necessary for closed shells are termed magic numbers. Clusters satisfying the magic number requirement have been detected and characterized by gas-phase spectroscopic methods. The most outstanding example is an aluminum cluster, Al_13_^−^, having *I*_h_ symmetry^4^. The molecular orbitals of this cluster are superatomic *S*, *P*, *D*, *F*, ⋯ orbitals and a stable closed-shell structure is formed similar to that of a halogen anion. Various other examples have also been reported, including Au_20_ having *T*_d_ symmetry^10^. The spherical jellium model has been applied with considerable success in cluster science. However, even if a cluster belongs to the highest point group, *I*_h_, its superatomic orbitals will split depending on their irreducible representations. The degree of degeneracy must be less than six based on point-group theory^11^, with the exception of spin-orbital degeneracy.

Non-geometrical symmetry has also been known, which gives rise to a greater degree of orbital degeneracy than that of spherical symmetry *O*(3)^3,12^. As this symmetry originates not from geometrical properties but rather from dynamical characteristics, it is referred to as dynamical symmetry. A typical example is the hydrogen atom, in which the unoccupied atomic orbitals are highly degenerate, because its Hamiltonian possesses symmetry associated with the Laplace–Runge–Lenz vector^13^, in addition to three-dimensional spherical symmetry. As a result, the atom formally possesses four-dimensional spherical symmetry. Another example is the three-dimensional isotropic harmonic oscillator, in which the 2*S* and 1*D* orbitals are 6-fold degenerate, the 2*P* and 1*F* orbitals are 10-fold degenerate, and the 3*S*, 2*D*, and 1*G* orbitals are 15-fold degenerate, and so on. This occurs because the Hamiltonian of this oscillator has *U*(3) symmetry^1,3,12,14^. With the exception of the extreme example of the hydrogen atom, such dynamical symmetry has not yet been found in an actual substance. However, it is expected that species with this type of symmetry could exceed the spherical symmetry of atoms and exhibit unique electronic and magnetic properties.

Here we demonstrate that realistic magnesium, zinc, and cadmium clusters having a specific tetrahedral framework possess anomalous higher-fold degeneracies than spherical symmetry from first principles. In addition, by means of simple tight-binding models and group-theoretical analyses, we elucidate that these degeneracies can be attributed to dynamical symmetry.

## Results

### First-principles calculations

**Fig. 1:**
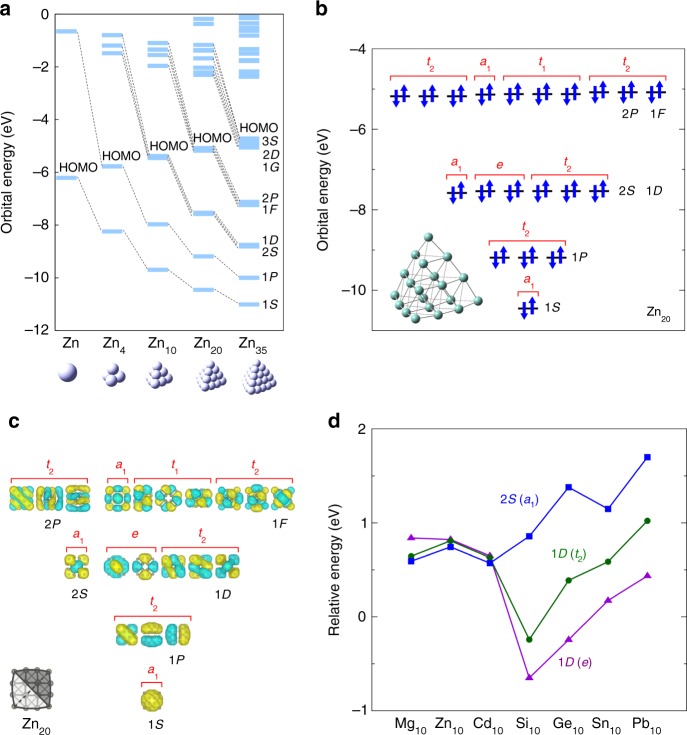
DFT calculations of tetrahedral clusters. **a** Molecular orbital levels of Zn_1_, Zn_4_, Zn_10_, Zn_20_, and Zn_35_. Zn_35_ favors a lower symmetry but was constrained to have *T*_d_ symmetry for comparison purposes. **b** Occupied molecular orbital levels of Zn_20_ with their irreducible representations and the optimized geometry. **c** Molecular orbitals of Zn_20_, the isosurface values of which are 1.0 × 10^−2^ a.u. **d** 2*S* and 1*D* molecular orbital levels of Mg_10_, Zn_10_, Cd_10_, Si_10_, Ge_10_, Sn_10_, and Pb_10_. The molecular orbital levels of each cluster have been shifted such that they originate at the atomic orbital level of the constitutive element. All the above calculations were carried out at the B3LYP/LanL2DZ level of theory implemented in Gaussian 09, Rev. E.01^39^

Density functional theory (DFT) calculations were performed for *T*_d_ symmetrical structures with valence electron numbers sufficient for the full occupation of superatomic orbitals. Figure 1a, b show the molecular orbital levels obtained for Zn_4_, Zn_10_, Zn_20_, and Zn_35_. Owing to their high geometrical symmetry, these orbitals can be ascribed to superatomic *S*, *P*, *D*, *F*, ⋯ orbitals, depending on the orbital angular momenta (Fig. 1c). A remarkable aspect of these results is that the occupied orbitals have unusual higher-fold degeneracies: the 2*S* and 1*D* orbitals are 6-fold degenerate, the 2*P* and 1*F* orbitals are 10-fold degenerate, and the 3*S*, 2*D*, and 1*G* orbitals are 15-fold degenerate. Such degeneracies are usually impossible, even in a spherical system. It should be noted that this degeneracy pattern is also consistent with that of the three-dimensional isotropic harmonic oscillator (Supplementary Fig. 1), as discussed further on. Equivalent degrees of degeneracy were also found in the case of other similar clusters, *X*_4_, *X*_10_, *X*_20_, and *X*_35_ (*X* = Mg, Cd), as shown in Supplementary Figs. 2 and 3. Prior theoretical studies of Mg, Zn, and Cd clusters have noted the closed-shell structures of these species^15–21^. These neutral clusters have never been experimentally detected due to the associated technical difficulties, although their charged clusters having similar atomicity have been reported^22–29^. Figure 1d plots the 2*S*(*a*_1_), 1*D*(*t*_2_), and 1*D*(*e*) molecular orbital levels of Mg_10_, Zn_10_, Cd_10_, Si_10_, Ge_10_, Sn_10_, and Pb_10_ (see also Supplementary Figs. 4 and 5). Although all the clusters have *T*_d_ symmetry, the degrees of level splitting are different. Mg_10_, Zn_10_, and Cd_10_ have higher-fold orbital degeneracies, whereas Si_10_, Ge_10_, Sn_10_, and Pb_10_ have greater degrees of level splitting. The reason for this difference is discussed further on. In addition to these homonuclear clusters, several heteronuclear clusters with higher-fold degeneracies were also found: Zn_6_Cd_4_ and Cd_6_Zn_4_ (Supplementary Fig. 6). In particular, Cd_6_Zn_4_ exhibits an extremely small energy difference of only 15 meV between its 1*D* and 2*S* orbitals (cf. 80 meV for Zn_10_). For comparison purposes, other heteronuclear clusters, including Al_6_Sn_4_, Ga_6_Sn_4_, and In_6_Sn_4_, are also shown in Supplementary Figs. 7 and 8. Among these, Al_6_Sn_4_ exhibits significant splitting of the 1*D* and 2*S* orbitals (1.218 eV).

### Tight-binding model analyses

**Fig. 2:**
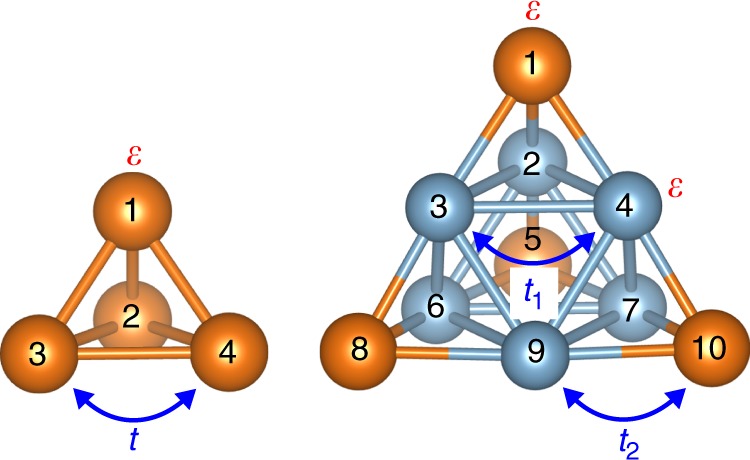
Model parameters for four-atom and ten-atom systems

**Fig. 3:**
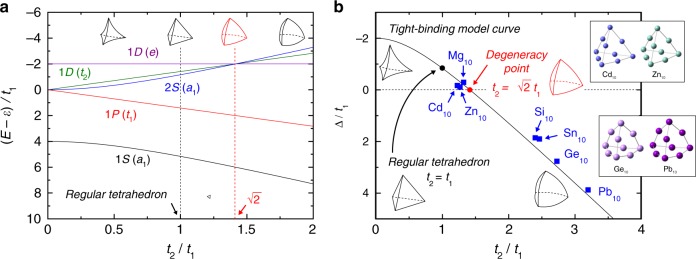
The tight-binding model analysis for the ten-atom tetrahedral system. **a** Energy spectrum of *H*_10_. **b** Degree of level splitting, Δ/*t*_1_, vs. the transfer integral ratio, *t*_2_/*t*_1_. Blue squares indicate the DFT value of each level splitting. All the parameters were determined based on DFT calculations at the B3LYP/LanL2DZ level of theory (Table 1). DFT-based optimized structures are also included

To interpret these results theoretically, simple tight-binding models^2,30,31^ were constructed and analyzed as follows (Fig. 2). For simplicity, only *s*-type valence atomic orbitals were taken into account. First, a tight-binding model Hamiltonian for a four-atom tetrahedral structure was constructed as

1 $$H_4 = \left( {\begin{array}{*{20}{c}} \varepsilon & t & t & t \\ t & \varepsilon & t & t \\ t & t & \varepsilon & t \\ t & t & t & \varepsilon \end{array}} \right),$$

where *ε* is an atomic orbital energy and *t* is a through-bond transfer integral (Fig. 2)^32^. The eigenvalues of *H*_4_ can be analytically obtained as *ε* + 3*t*, *ε* − *t*, *ε* − *t*, *ε* − *t*, corresponding to the 1*S*(*a*_1_) and 1*P*(*t*_1_) levels, respectively. A threefold degeneracy associated with the 1*P* levels appears, which is trivial within point-group theory. Second, a tight-binding model Hamiltonian for a ten-atom tetrahedral structure was constructed as

2 $$H_{10} = \left( {\begin{array}{*{20}{l}} \varepsilon \hfill & {t_2} \hfill & {t_2} \hfill & {t_2} \hfill & 0 \hfill & 0 \hfill & 0 \hfill & 0 \hfill & 0 \hfill & 0 \hfill \\ {t_2} \hfill & \varepsilon \hfill & {t_1} \hfill & {t_1} \hfill & {t_2} \hfill & {t_1} \hfill & {t_1} \hfill & 0 \hfill & 0 \hfill & 0 \hfill \\ {t_2} \hfill & {t_1} \hfill & \varepsilon \hfill & {t_1} \hfill & 0 \hfill & {t_1} \hfill & 0 \hfill & {t_2} \hfill & {t_1} \hfill & 0 \hfill \\ {t_2} \hfill & {t_1} \hfill & {t_1} \hfill & \varepsilon \hfill & 0 \hfill & 0 \hfill & {t_1} \hfill & 0 \hfill & {t_1} \hfill & {t_2} \hfill \\ 0 \hfill & {t_2} \hfill & 0 \hfill & 0 \hfill & \varepsilon \hfill & {t_2} \hfill & {t_2} \hfill & 0 \hfill & 0 \hfill & 0 \hfill \\ 0 \hfill & {t_1} \hfill & {t_1} \hfill & 0 \hfill & {t_2} \hfill & \varepsilon \hfill & {t_1} \hfill & {t_2} \hfill & {t_1} \hfill & 0 \hfill \\ 0 \hfill & {t_1} \hfill & 0 \hfill & {t_1} \hfill & {t_2} \hfill & {t_1} \hfill & \varepsilon \hfill & 0 \hfill & {t_1} \hfill & {t_2} \hfill \\ 0 \hfill & 0 \hfill & {t_2} \hfill & 0 \hfill & 0 \hfill & {t_2} \hfill & 0 \hfill & \varepsilon \hfill & {t_2} \hfill & 0 \hfill \\ 0 \hfill & 0 \hfill & {t_1} \hfill & {t_1} \hfill & 0 \hfill & {t_1} \hfill & {t_1} \hfill & {t_2} \hfill & \varepsilon \hfill & {t_2} \hfill \\ 0 \hfill & 0 \hfill & 0 \hfill & {t_2} \hfill & 0 \hfill & 0 \hfill & {t_2} \hfill & 0 \hfill & {t_2} \hfill & \varepsilon \hfill \end{array}} \right),$$

where *t*_1_ and *t*_2_ are transfer integrals corresponding to different types of bonds (Fig. 2). It should be noted that the absolute value of a transfer integral increases as an interatomic distance decreases (Supplementary Fig. 9). The analytical eigenvalues were obtained as *ε* + 2*t*_1_ − $\sqrt {4t_1^2 + 6t_2^2}$, $\varepsilon + \sqrt 2 t_2$, $\varepsilon + \sqrt 2 t_2$, $\varepsilon + \sqrt 2 t_2$, *ε* + 2*t*_1_ + $\sqrt {4t_1^2 + 6t_2^2}$, *ε* − $\sqrt 2 t_2$, $\varepsilon - \sqrt 2 t_2$, $\varepsilon - \sqrt 2 t_2$, *ε* − 2*t*_1_, *ε* − 2*t*_1_, corresponding to the 1*S*(*a*_1_), 1*P*(*t*_1_), 2*S*(*a*_1_), 1*D*(*t*_2_), and 1*D*(*e*) levels, respectively. As illustrated in Fig. 3a, an unexpected degeneracy point associated with 1*D*(*t*_2_), 1*D*(*e*), and 2*S*(*a*_1_) appears. The condition producing this degeneracy was found not to be a regular tetrahedron (*t*_1_ = *t*_2_) but rather an inflated tetrahedron $\left( {t_2 = \sqrt 2 t_1} \right)$, and this simple *t*_2_-to-*t*_1_ ratio implies the existence of hidden symmetry. However, this abnormal degeneracy cannot be explained, at least within point-group theory, because transfer integral ratios corresponding to non-equivalent bonds are outside the scope of the theory.

The validity of the simple ten-atom system model was subsequently examined. Table 1 lists the model parameters (that is, *t*_1_ and *t*_2_) obtained from the DFT calculations. These values were estimated as half the level of splitting between bonding and anti-bonding orbitals of *s*-type valence electrons. The energy difference, Δ, between the 2*S* and 1*D* molecular orbitals can be calculated from the simple tight-binding model (Δ = −4*t*_1_ − $\sqrt {4t_1^2 + 6t_2^2}$). In addition, Δ can also be determined directly from DFT calculations (Table 1). The validity of the present model can be ascertained by comparing these values. Figure 3b plots the degree of level splitting, Δ/*t*_1_, as a function of the transfer integral ratio, *t*_2_/*t*_1_. It is evident that the DFT-based values (blue squares) perfectly follow the simple model curve. Thus, the present tight-binding model employing solely through-bond transfer integrals is in very good agreement with the DFT calculations. This agreement is attributed to the present system being constructed of three-simplexes, or four-atom tetrahedrons, which has few through-space interactions^32^ (see also Supplementary Note 1). Remarkably, Mg_10_, Zn_10_, and Cd_10_ are all located very close to an ideal degeneracy point $\left( {t_2 = \sqrt 2 t_1} \right)$, in contrast to Si_10_, Ge_10_, Sn_10_, and Pb_10_. This result explains why the degrees of level splitting vary between different elements.

**Table 1:** The evaluated parameters by DFT calculations

	Mg_10_	Zn_10_	Cd_10_	Si_10_	Ge_10_	Sn_10_	Pb_10_
*t*_1_ (eV)	− 0.85	− 0.72	− 0.46	− 0.81	− 0.59	− 0.51	− 0.33
*t*_2_ (eV)	− 1.12	− 0.91	− 0.56	− 1.95	− 1.60	− 1.26	− 1.04
Δ_TB_ (eV)	0.17	0.22	0.18	− 1.79	− 1.74	− 1.21	− 1.33
Δ_DFT_ (eV)	0.25	0.08	0.08	− 1.51	− 1.62	− 0.97	− 1.26

Δ denotes the 1*D*(*e*) energy minus that of 2*S*(*a*_1_). Δ_TB_ and Δ_DFT_ were evaluated by the tight-binding model $\left( { - 4t_1 - \sqrt {4t_1^2 + 6t_2^2} } \right)$ and directly by DFT calculations, respectively. The B3LYP/LanL2DZ level of theory was employed for the DFT calculations

**Fig. 4:**
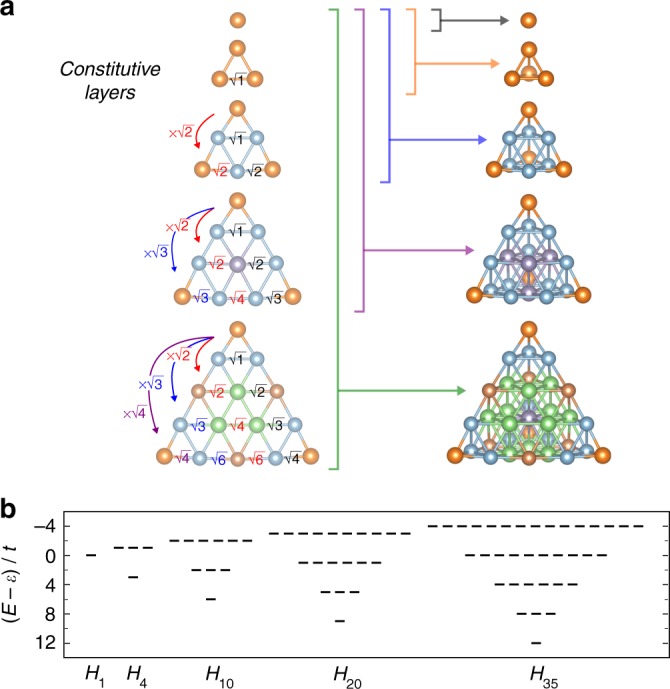
The tight-binding model analyses for tetrahedral clusters with various sizes. **a** Ratios of transfer integrals giving rise to the anomalous degeneracy, in which a square-root mathematical sequence appears. **b** Energy spectra of the tight-binding models under the degeneracy conditions. Here, *t* denotes a transfer integral whose absolute value is the smallest in each model

In the same manner, tight-binding model Hamiltonians for larger systems were also constructed (Supplementary Notes 2 and 3; Supplementary Figs. 10 and 11). A general condition for the anomalous orbital degeneracy was subsequently fully identified, as shown in Fig. 4a. Surprisingly, the degeneracy condition can be represented as an elegant square-root mathematical sequence (appearing in the so-called spiral of Theodorus) involving the ratio of transfer integrals. In each case, the degeneracy condition corresponds not to a regular tetrahedron but rather to an inflated tetrahedron.

## Discussion

To elucidate the cause of the higher-fold degeneracies, the symmetry of each model was analyzed. The Hamiltonian, *H*_*N*_ (*N* = 1, 4, 10, 20, ⋯), satisfying the degeneracy condition can be rewritten as

3 $$H_N = \left\{ {\varepsilon + 3\left( {n - 1} \right)t} \right\}I - 4t\mathop {\sum}\limits_{i = 1}^3 {\kern 1pt} a_i^\dagger a_i,$$

where *n* is the number of constitutive layers in the *N*-atom tetrahedral system, *I* is the identity matrix, and $a_i^\dagger$ and *a*_*i*_ (*i* = 1, 2, 3) are creation and annihilation operators on *N*-dimensional Hilbert space. Thus, the present system is equivalent to the harmonic oscillator with *U*(3) symmetry under the degeneracy condition. In fact, *H*_N_ is invariant under the following transformations,

4 $$a_i \mapsto \mathop {\sum}\limits_{j = 1}^3 {\kern 1pt} u_{ij}a_j,\quad a_i^\dagger \mapsto \mathop {\sum}\limits_{k = 1}^3 {\kern 1pt} u_{ik}^ \ast a_k^\dagger \quad (i = 1,2,3),$$

where the unitary condition is satisfied as

5 $$\mathop {\sum}\limits_{i = 1}^3 {\kern 1pt} u_{ik}^ \ast u_{ij} = \delta _{kj}.$$

The energy spectra obtained under the degeneracy conditions are equivalent to that of the harmonic oscillator, as shown in Fig. 4b. This equality is believed to result from the coincidence of atomicity in the *n*th layer of the tetrahedron and the orbital degeneracy of the *n*th energy of the harmonic oscillator (Supplementary Note 4; Supplementary Figs. 12 and 13). The group chain *U*(3) ⊃ *O*(3) indicates that the present system has a higher degree of symmetry than the highest geometrical symmetry.

In general, all metal clusters cannot have *O*(3), because they have finite numbers of constitutive atoms in their geometrical structures. Therefore, it is just an approximative picture that some magic clusters satisfy *O*(3). This is the shell model^9^, in which geometrical information is all abstracted and instead a spherical structure is assumed for simplicity. In contrast to such a traditional view, the present work demonstrates that tetrahedral clusters are able to satisfy the dynamical symmetry higher than *O*(3) exactly. This is notable because geometrical abstraction is not necessary any more. Figure 3a illustrates that the energy of 1*D*(*t*_2_) is closer to that of 2*S*(*a*_1_) than to that of 1*D*(*e*), because the 1*D*(*t*_2_) and 2*S*(*a*_1_) orbitals have distributions on the four vertex atoms, whereas the 1*D*(*e*) orbitals have no distribution at the vertices (see Supplementary Fig. 14). This is the intrinsic orbital splitting pattern of tetrahedral clusters. As a result, the degeneracy of 1*D*(*t*_2_) and 1*D*(*e*) does not appear independently of 2*S*(*a*_1_). Instead, 1*D*(*t*_2_), 1*D*(*e*), and 2*S*(*a*_1_) are all degenerate just at a triple point. This implies that tetrahedral clusters achieve the dynamical symmetry not via *O*(3). On the other hand, in the case of tetrahedral clusters being too inflated and quasi-spherical, 1*D*(*t*_2_) pairs with 1*D*(*e*) rather than with 2*S*(*a*_1_), as shown in Fig. 1d.

A prior theoretical study has reported that the spherical jellium model spontaneously deforms in a tetrahedral direction, if its shape is relaxed under the condition that the total number of valence electrons coincides with the magic number of the three-dimensional isotropic harmonic oscillator^15^. The present study illustrates the reason why tetrahedrally deformed orbitals are special by identifying the exact *U*(3) point in the tetrahedral frameworks on the basis of the simple tight-binding models.

In conclusion, nanomaterials that surpass the symmetry of spherical atoms can be realized by considering not only geometrical symmetry but also dynamical symmetry. Owing to this nontrivial symmetry, these species have super-degenerate molecular orbitals that give rise to an extremely high discrete density of states around the Fermi level. Carrier doping into these super-degenerate orbitals could lead to excellent electric conductivity, as long as the unfavorable Jahn–Teller effect is not so considerable. In addition, the spin arrangement in super-degenerate orbitals could yield unique magnetism. Unlike atoms, it is impossible to realize arbitrary spin states, because of the Jahn–Teller effect. However, a certain high spin state can be stabilized by matching the number of singly-occupying electrons with the degree of super-degeneracy through alloying or changing a constitutive element (see Supplementary Figs. 15 and 16). Such a state could be obtained by synthesizing clusters under a magnetic field. Thus, the control of dynamical symmetry should lead to the development of next-generation electronic and magnetic materials.

The proposed clusters are to be viable^33^ in light of prior experimental studies. The laser vaporization techniques combined with time-of-flight mass spectrometry produced Mg, Zn, Cd clusters with tetrahedral atomicities in the gas phase^22–29^. A soft-landing method  onto self-assembled monolayers should be effective to obtain the clusters as materials^34^. As for the liquid phase, a template-based method can be used to fabricate the clusters^35^. Ligand protection might realize the isolation and crystallization^36^. It is also helpful to refer to the crystallization of Zintl clusters^37^. Furthermore, much attention should be paid to emerging cocrystallization techniques of superatomic clusters and fullerenes^38^. Some of these synthetic methods should be suitable for the proposed clusters. The present study features Mg, Zn, and Cd tetrahedral clusters. However, other tetrahedral clusters with inappropriate structures for super-degeneracy, being too inflated, are also of interest under mechanical pressure.

## Methods

### First-principles calculations

By employing the DFT method, geometry optimizations and vibrational analyses were performed for *T*_d_ symmetrical structures with valence electron numbers sufficient for the full occupation of superatomic orbitals. Specifically, the homonuclear clusters *X*_4_, *X*_10_, *X*_20_, and *X*_35_ (*X* = Mg, Zn, Cd) and the heteronuclear clusters *X*_6_*Y*_4_ (*X*, *Y* = Zn, Cd) were calculated. These clusters have 8, 20, 40, 70, and 20 *s*-type valence electrons, respectively. For comparison purposes, the 40 valence electron systems Si_10_, Ge_10_, Sn_10_, Pb_10_, Al_6_Sn_4_, Ga_6_Sn_4_, and In_6_Sn_4_, the 14 valence electron system Au_6_Zn_4_, and the 30 valence electron system Tl_10_ were also calculated. All the DFT calculations were conducted with the B3LYP functional and LanL2DZ basis set, using the Gaussian 09, Rev. E.01 program package^39^. All the structure data are available in Supplementary Tables 1–23.

## Electronic supplementary material

Supplementary Information

## Data Availability

The data that support the findings of this study are available from the corresponding author upon reasonable request.
