# Supplementary material for: Nanomaterials design for super-degenerate electronic state beyond the limit of geometrical symmetry
Source: Nat Commun. 2018 Sep 14;9:3758. doi: 10.1038/s41467-018-06244-8 (PMC6138727; doi:10.1038/s41467-018-06244-8)
Supplement: Supplementary file 1 — Supplementary Information [file 41467_2018_6244_MOESM1_ESM.pdf]

Supplementary Information

Nanomaterials Design for Super-Degenerate Electronic State Beyond the Limit of Geometrical Symmetry

Haruta *et al.*

Supplementary Note 1 Through-Space Interactions

In the DFT calculations, the orbital splitting of the Mg clusters in eV is slightly greater than those of the Zn and Cd clusters, partially due to through-space interactions. For example, a 10-atom tetrahedral cluster has only one type of through-space interaction inside the central blue-colored octahedron in Fig. 2. Such an interaction only slightly violates the simple tight-binding picture. The magnitude of the through-space transfer integrals has the following order: Mg_{10} (-0.23 eV) $>$ Zn_{10} (-0.16 eV) $>$ Cd_{10} (-0.08 eV), depending on the number of total electrons of each element, which affects its interatomic distance and the size of its outermost atomic orbital. That is the reason for the greater orbital splitting of the Mg clusters, which is negligible as compared with many other clusters.

Supplementary Note 2 Tight-Binding Model for 20-Atom System

The tight-binding model Hamiltonian of the 20-atom structure can be constructed as

$$H_{20} = \begin{pmatrix}
\varepsilon & t_2 & t_2 & t_2 & 0 & 0 & 0 & 0 & 0 & 0 & 0 & 0 & 0 & 0 & 0 & 0 & 0 & 0 & 0 & 0 \\
t_2 & \varepsilon & t_4 & t_4 & t_1 & t_3 & t_3 & 0 & 0 & 0 & 0 & 0 & 0 & 0 & 0 & 0 & 0 & 0 & 0 & 0 \\
t_2 & t_4 & \varepsilon & t_4 & 0 & t_3 & 0 & t_1 & t_3 & 0 & 0 & 0 & 0 & 0 & 0 & 0 & 0 & 0 & 0 & 0 \\
t_2 & t_4 & t_4 & \varepsilon & 0 & 0 & t_3 & 0 & t_3 & t_1 & 0 & 0 & 0 & 0 & 0 & 0 & 0 & 0 & 0 & 0 \\
0 & t_1 & 0 & 0 & \varepsilon & t_3 & t_3 & 0 & 0 & 0 & t_2 & t_4 & t_4 & 0 & 0 & 0 & 0 & 0 & 0 & 0 \\
0 & t_3 & t_3 & 0 & t_3 & \varepsilon & t_5 & t_3 & t_5 & 0 & 0 & t_3 & 0 & t_3 & t_5 & 0 & 0 & 0 & 0 & 0 \\
0 & t_3 & 0 & t_3 & t_3 & t_5 & \varepsilon & 0 & t_5 & t_3 & 0 & 0 & t_3 & 0 & t_5 & t_3 & 0 & 0 & 0 & 0 \\
0 & 0 & t_1 & 0 & 0 & t_3 & 0 & \varepsilon & t_3 & 0 & 0 & 0 & 0 & t_4 & 0 & 0 & t_2 & t_4 & 0 & 0 \\
0 & 0 & t_3 & t_3 & 0 & t_5 & t_5 & t_3 & \varepsilon & t_3 & 0 & 0 & 0 & 0 & t_5 & 0 & 0 & t_3 & t_3 & 0 \\
0 & 0 & 0 & t_1 & 0 & 0 & t_3 & 0 & t_3 & \varepsilon & 0 & 0 & 0 & 0 & 0 & t_4 & 0 & 0 & t_4 & t_2 \\
0 & 0 & 0 & 0 & t_2 & 0 & 0 & 0 & 0 & 0 & \varepsilon & t_2 & t_2 & 0 & 0 & 0 & 0 & 0 & 0 & 0 \\
0 & 0 & 0 & 0 & t_4 & t_3 & 0 & 0 & 0 & 0 & t_2 & \varepsilon & t_4 & t_1 & t_3 & 0 & 0 & 0 & 0 & 0 \\
0 & 0 & 0 & 0 & t_4 & 0 & t_3 & 0 & 0 & 0 & t_2 & t_4 & \varepsilon & 0 & t_3 & t_1 & 0 & 0 & 0 & 0 \\
0 & 0 & 0 & 0 & 0 & t_3 & 0 & t_4 & 0 & 0 & 0 & t_1 & 0 & \varepsilon & t_3 & 0 & t_2 & t_4 & 0 & 0 \\
0 & 0 & 0 & 0 & 0 & t_5 & t_5 & 0 & t_5 & 0 & 0 & t_3 & t_3 & t_3 & \varepsilon & t_3 & 0 & t_3 & t_3 & 0 \\
0 & 0 & 0 & 0 & 0 & 0 & t_3 & 0 & 0 & t_4 & 0 & 0 & t_1 & 0 & t_3 & \varepsilon & 0 & 0 & t_4 & t_2 \\
0 & 0 & 0 & 0 & 0 & 0 & 0 & t_2 & 0 & 0 & 0 & 0 & 0 & t_2 & 0 & 0 & \varepsilon & t_2 & 0 & 0 \\
0 & 0 & 0 & 0 & 0 & 0 & 0 & t_4 & t_3 & 0 & 0 & 0 & 0 & t_4 & t_3 & 0 & t_2 & \varepsilon & t_1 & 0 \\
0 & 0 & 0 & 0 & 0 & 0 & 0 & 0 & t_3 & t_4 & 0 & 0 & 0 & 0 & t_3 & t_4 & 0 & t_1 & \varepsilon & t_2 \\
0 & 0 & 0 & 0 & 0 & 0 & 0 & 0 & 0 & t_2 & 0 & 0 & 0 & 0 & 0 & t_2 & 0 & 0 & t_2 & \varepsilon
\end{pmatrix} \tag{1}$$

where ε is an atomic orbital energy and t_i ($i = 1, \cdots, 5$) are transfer integrals corresponding to different types of bonds (Supplementary Fig. 10). The eigenvalues of H_{20} are obtained as $\varepsilon + 9t_4$, $\varepsilon + 5t_4$, $\varepsilon + 5t_4$, $\varepsilon + 5t_4$, $\varepsilon + t_4$, $\varepsilon + t_4$, $\varepsilon + t_4$, $\varepsilon + t_4$, $\varepsilon + t_4$, $\varepsilon + t_4$, $\varepsilon - 3t_4$, $\varepsilon - 3t_4$, $\varepsilon - 3t_4$, $\varepsilon - 3t_4$, $\varepsilon - 3t_4$, $\varepsilon - 3t_4$, $\varepsilon - 3t_4$, $\varepsilon - 3t_4$, $\varepsilon - 3t_4$, $\varepsilon - 3t_4$ under the following special conditions,

$$t_1 = \sqrt{4}t_4, \quad t_2 = \sqrt{3}t_4, \quad t_3 = \sqrt{2}t_4, \quad t_5 = \sqrt{1}t_4. \tag{2}$$

These energy levels correspond to $1S$, $1P$, $(1D, 2S)$, $(1F, 2P)$ molecular orbitals, respectively.

Supplementary Note 3 Tight-Binding Model for 35-Atom System

The tight-binding model Hamiltonian of the 35-atom structure can be constructed as

$$
H_{35} =
\begin{pmatrix}
\varepsilon & t_1 & t_1 & t_1 & 0 & 0 & 0 & 0 & 0 & 0 & 0 & 0 & 0 & 0 & 0 & 0 & 0 & 0 & 0 & 0 & 0 & 0 & 0 & 0 & 0 & 0 & 0 & 0 & 0 & 0 & 0 & 0 & 0 & 0 & 0 \\
t_1 & \varepsilon & t_5 & t_5 & t_2 & t_3 & t_3 & 0 & 0 & 0 & 0 & 0 & 0 & 0 & 0 & 0 & 0 & 0 & 0 & 0 & 0 & 0 & 0 & 0 & 0 & 0 & 0 & 0 & 0 & 0 & 0 & 0 & 0 & 0 & 0 \\
t_1 & t_5 & \varepsilon & t_5 & 0 & t_3 & 0 & t_2 & t_3 & 0 & 0 & 0 & 0 & 0 & 0 & 0 & 0 & 0 & 0 & 0 & 0 & 0 & 0 & 0 & 0 & 0 & 0 & 0 & 0 & 0 & 0 & 0 & 0 & 0 & 0 \\
t_1 & t_5 & t_5 & \varepsilon & 0 & 0 & t_3 & 0 & t_3 & t_2 & 0 & 0 & 0 & 0 & 0 & 0 & 0 & 0 & 0 & 0 & 0 & 0 & 0 & 0 & 0 & 0 & 0 & 0 & 0 & 0 & 0 & 0 & 0 & 0 & 0 \\
0 & t_2 & 0 & 0 & \varepsilon & t_6 & t_6 & 0 & 0 & 0 & t_2 & t_6 & t_6 & 0 & 0 & 0 & 0 & 0 & 0 & 0 & 0 & 0 & 0 & 0 & 0 & 0 & 0 & 0 & 0 & 0 & 0 & 0 & 0 & 0 & 0 \\
0 & t_3 & t_3 & 0 & t_6 & \varepsilon & t_8 & t_6 & t_8 & 0 & 0 & t_4 & 0 & t_4 & t_7 & 0 & 0 & 0 & 0 & 0 & 0 & 0 & 0 & 0 & 0 & 0 & 0 & 0 & 0 & 0 & 0 & 0 & 0 & 0 & 0 \\
0 & t_3 & 0 & t_3 & t_6 & t_8 & \varepsilon & 0 & t_8 & t_6 & 0 & 0 & t_4 & 0 & t_7 & t_4 & 0 & 0 & 0 & 0 & 0 & 0 & 0 & 0 & 0 & 0 & 0 & 0 & 0 & 0 & 0 & 0 & 0 & 0 & 0 \\
0 & 0 & t_2 & 0 & 0 & t_6 & 0 & \varepsilon & t_6 & 0 & 0 & 0 & 0 & t_6 & 0 & 0 & t_2 & t_6 & 0 & 0 & 0 & 0 & 0 & 0 & 0 & 0 & 0 & 0 & 0 & 0 & 0 & 0 & 0 & 0 & 0 \\
0 & 0 & t_3 & t_3 & 0 & t_8 & t_8 & t_6 & \varepsilon & t_6 & 0 & 0 & 0 & 0 & t_7 & 0 & 0 & t_4 & t_4 & 0 & 0 & 0 & 0 & 0 & 0 & 0 & 0 & 0 & 0 & 0 & 0 & 0 & 0 & 0 & 0 \\
0 & 0 & 0 & t_2 & 0 & 0 & t_6 & 0 & t_6 & \varepsilon & 0 & 0 & 0 & 0 & 0 & t_6 & 0 & 0 & t_6 & t_2 & 0 & 0 & 0 & 0 & 0 & 0 & 0 & 0 & 0 & 0 & 0 & 0 & 0 & 0 & 0 \\
0 & 0 & 0 & 0 & t_2 & 0 & 0 & 0 & 0 & 0 & \varepsilon & t_3 & t_3 & 0 & 0 & 0 & 0 & 0 & 0 & 0 & 0 & 0 & 0 & 0 & 0 & 0 & 0 & 0 & t_5 & t_5 & t_1 & 0 & 0 & 0 & 0 \\
0 & 0 & 0 & 0 & t_6 & t_4 & 0 & 0 & 0 & 0 & t_3 & \varepsilon & t_8 & t_4 & t_7 & 0 & 0 & 0 & 0 & 0 & 0 & 0 & 0 & 0 & t_8 & t_6 & 0 & t_3 & 0 & 0 & 0 & 0 & 0 & 0 & 0 \\
0 & 0 & 0 & 0 & t_6 & 0 & t_4 & 0 & 0 & 0 & t_3 & t_8 & \varepsilon & 0 & t_7 & t_4 & 0 & 0 & 0 & 0 & 0 & 0 & 0 & 0 & t_8 & 0 & t_6 & 0 & t_3 & 0 & 0 & 0 & 0 & 0 & 0 \\
0 & 0 & 0 & 0 & 0 & t_4 & 0 & t_6 & 0 & 0 & 0 & t_4 & 0 & \varepsilon & t_7 & 0 & t_3 & t_8 & 0 & 0 & t_8 & 0 & t_3 & 0 & 0 & t_6 & 0 & 0 & 0 & 0 & 0 & 0 & 0 & 0 & 0 \\
0 & 0 & 0 & 0 & 0 & t_7 & t_7 & 0 & t_7 & 0 & 0 & t_7 & t_7 & t_7 & \varepsilon & t_7 & 0 & t_7 & t_7 & 0 & t_7 & t_7 & 0 & 0 & t_7 & 0 & 0 & 0 & 0 & 0 & 0 & 0 & 0 & 0 & 0 \\
0 & 0 & 0 & 0 & 0 & 0 & t_4 & 0 & 0 & t_6 & 0 & 0 & t_4 & 0 & t_7 & \varepsilon & 0 & 0 & t_8 & t_3 & 0 & t_8 & 0 & t_3 & 0 & 0 & t_6 & 0 & 0 & 0 & 0 & 0 & 0 & 0 & 0 \\
0 & 0 & 0 & 0 & 0 & 0 & 0 & t_2 & 0 & 0 & 0 & 0 & 0 & t_3 & 0 & 0 & \varepsilon & t_3 & 0 & 0 & 0 & 0 & t_5 & 0 & 0 & 0 & 0 & 0 & 0 & 0 & t_1 & t_5 & 0 & 0 & 0 \\
0 & 0 & 0 & 0 & 0 & 0 & 0 & t_6 & t_4 & 0 & 0 & 0 & 0 & t_8 & t_7 & 0 & t_3 & \varepsilon & t_4 & 0 & t_8 & 0 & 0 & 0 & 0 & 0 & 0 & 0 & 0 & 0 & 0 & t_3 & t_6 & 0 & 0 \\
0 & 0 & 0 & 0 & 0 & 0 & 0 & 0 & t_4 & t_6 & 0 & 0 & 0 & 0 & t_7 & t_8 & 0 & t_4 & \varepsilon & t_3 & 0 & t_8 & 0 & 0 & 0 & 0 & 0 & 0 & 0 & 0 & 0 & 0 & t_6 & t_3 & 0 \\
0 & 0 & 0 & 0 & 0 & 0 & 0 & 0 & 0 & t_2 & 0 & 0 & 0 & 0 & 0 & t_3 & 0 & 0 & t_3 & \varepsilon & 0 & 0 & 0 & t_5 & 0 & 0 & 0 & 0 & 0 & 0 & 0 & 0 & 0 & t_5 & t_1 \\
0 & 0 & 0 & 0 & 0 & 0 & 0 & 0 & 0 & 0 & 0 & 0 & 0 & t_8 & t_7 & 0 & 0 & t_8 & 0 & 0 & \varepsilon & t_4 & t_3 & 0 & t_4 & t_6 & 0 & 0 & 0 & 0 & 0 & t_3 & t_6 & 0 & 0 \\
0 & 0 & 0 & 0 & 0 & 0 & 0 & 0 & 0 & 0 & 0 & 0 & 0 & 0 & t_7 & t_8 & 0 & 0 & t_8 & 0 & t_4 & \varepsilon & 0 & t_3 & t_4 & 0 & t_6 & 0 & 0 & 0 & 0 & 0 & t_6 & t_3 & 0 \\
0 & 0 & 0 & 0 & 0 & 0 & 0 & 0 & 0 & 0 & 0 & 0 & 0 & t_3 & 0 & 0 & t_5 & 0 & 0 & 0 & t_3 & 0 & \varepsilon & 0 & 0 & t_2 & 0 & 0 & 0 & 0 & t_1 & t_5 & 0 & 0 & 0 \\
0 & 0 & 0 & 0 & 0 & 0 & 0 & 0 & 0 & 0 & 0 & 0 & 0 & 0 & 0 & t_3 & 0 & 0 & 0 & t_5 & 0 & t_3 & 0 & \varepsilon & 0 & 0 & t_2 & 0 & 0 & 0 & 0 & 0 & 0 & t_5 & t_1 \\
0 & 0 & 0 & 0 & 0 & 0 & 0 & 0 & 0 & 0 & 0 & t_8 & t_8 & 0 & t_7 & 0 & 0 & 0 & 0 & 0 & t_4 & t_4 & 0 & 0 & \varepsilon & t_6 & t_6 & t_3 & t_3 & 0 & 0 & 0 & 0 & 0 & 0 \\
0 & 0 & 0 & 0 & 0 & 0 & 0 & 0 & 0 & 0 & 0 & t_6 & 0 & t_6 & 0 & 0 & 0 & 0 & 0 & 0 & t_6 & 0 & t_2 & 0 & t_6 & \varepsilon & 0 & t_2 & 0 & 0 & 0 & 0 & 0 & 0 & 0 \\
0 & 0 & 0 & 0 & 0 & 0 & 0 & 0 & 0 & 0 & 0 & 0 & t_6 & 0 & 0 & t_6 & 0 & 0 & 0 & 0 & 0 & t_6 & 0 & t_2 & t_6 & 0 & \varepsilon & 0 & t_2 & 0 & 0 & 0 & 0 & 0 & 0 \\
0 & 0 & 0 & 0 & 0 & 0 & 0 & 0 & 0 & 0 & t_5 & t_3 & 0 & 0 & 0 & 0 & 0 & 0 & 0 & 0 & 0 & 0 & 0 & 0 & t_3 & t_2 & 0 & \varepsilon & t_5 & t_1 & 0 & 0 & 0 & 0 & 0 \\
0 & 0 & 0 & 0 & 0 & 0 & 0 & 0 & 0 & 0 & t_5 & 0 & t_3 & 0 & 0 & 0 & 0 & 0 & 0 & 0 & 0 & 0 & 0 & 0 & t_3 & 0 & t_2 & t_5 & \varepsilon & t_1 & 0 & 0 & 0 & 0 & 0 \\
0 & 0 & 0 & 0 & 0 & 0 & 0 & 0 & 0 & 0 & t_1 & 0 & 0 & 0 & 0 & 0 & 0 & 0 & 0 & 0 & 0 & 0 & 0 & 0 & 0 & 0 & 0 & t_1 & t_1 & \varepsilon & 0 & 0 & 0 & 0 & 0 \\
0 & 0 & 0 & 0 & 0 & 0 & 0 & 0 & 0 & 0 & 0 & 0 & 0 & 0 & 0 & 0 & t_1 & 0 & 0 & 0 & 0 & 0 & t_1 & 0 & 0 & 0 & 0 & 0 & 0 & 0 & \varepsilon & t_1 & 0 & 0 & 0 \\
0 & 0 & 0 & 0 & 0 & 0 & 0 & 0 & 0 & 0 & 0 & 0 & 0 & 0 & 0 & 0 & t_5 & t_3 & 0 & 0 & t_3 & 0 & t_5 & 0 & 0 & 0 & 0 & 0 & 0 & 0 & t_1 & \varepsilon & t_2 & 0 & 0 \\
0 & 0 & 0 & 0 & 0 & 0 & 0 & 0 & 0 & 0 & 0 & 0 & 0 & 0 & 0 & 0 & 0 & t_6 & t_6 & 0 & t_6 & t_6 & 0 & 0 & 0 & 0 & 0 & 0 & 0 & 0 & 0 & t_2 & \varepsilon & t_2 & 0 \\
0 & 0 & 0 & 0 & 0 & 0 & 0 & 0 & 0 & 0 & 0 & 0 & 0 & 0 & 0 & 0 & 0 & 0 & t_3 & t_5 & 0 & t_3 & 0 & t_5 & 0 & 0 & 0 & 0 & 0 & 0 & 0 & 0 & t_2 & \varepsilon & t_1 \\
0 & 0 & 0 & 0 & 0 & 0 & 0 & 0 & 0 & 0 & 0 & 0 & 0 & 0 & 0 & 0 & 0 & 0 & 0 & t_1 & 0 & 0 & 0 & t_1 & 0 & 0 & 0 & 0 & 0 & 0 & 0 & 0 & 0 & t_1 & \varepsilon
\end{pmatrix}, \tag{3}
$$

where ε is an atomic orbital energy and t_i ($i = 1, \cdots, 8$) are transfer integrals corresponding to different types of bonds (Supplementary Fig. 11). The eigenvalues of H_{35} are obtained as $\varepsilon + 12t_5$, $\varepsilon + 8t_5$, $\varepsilon + 8t_5$, $\varepsilon + 8t_5$, $\varepsilon + 4t_5$, $\varepsilon + 4t_5$, $\varepsilon + 4t_5$, $\varepsilon + 4t_5$, $\varepsilon + 4t_5$, $\varepsilon + 4t_5$, ε, ε, ε, ε, ε, ε, ε, ε, ε, ε, $\varepsilon - 4t_5$, $\varepsilon - 4t_5$, $\varepsilon - 4t_5$, $\varepsilon - 4t_5$, $\varepsilon - 4t_5$, $\varepsilon - 4t_5$, $\varepsilon - 4t_5$, $\varepsilon - 4t_5$, $\varepsilon - 4t_5$, $\varepsilon - 4t_5$, $\varepsilon - 4t_5$, $\varepsilon - 4t_5$, $\varepsilon - 4t_5$, $\varepsilon - 4t_5$, $\varepsilon - 4t_5$ under the following special conditions,

$$t_1 = \sqrt{4}t_5, \quad t_2 = \sqrt{6}t_5, \quad t_3 = \sqrt{3}t_5, \quad t_4 = \sqrt{4}t_5, \quad t_6 = \sqrt{2}t_5, \quad t_7 = \sqrt{2}t_5, \quad t_8 = \sqrt{1}t_5. \tag{4}$$

These energy levels correspond to $1S$, $1P$, $(1D, 2S)$, $(1F, 2P)$, $(1G, 2D, 3S)$ molecular orbitals, respectively.

Supplementary Note 4 Tetrahedral Atomicity and Number of Eigenstates of 3D Harmonic Oscillator

As shown in Supplementary Fig. 12, an eigenenergy E of the 3-dimensional isotropic harmonic oscillator can be characterized by 3 vibrational quantum numbers n_x, n_y, n_z as

$$E = E(n_x, n_y, n_z) = \left(n_x + n_y + n_z + \frac{3}{2}\right)\hbar\omega. \tag{5}$$

Each eigenstate has the energy of $\frac{3}{2}\hbar\omega$, $\frac{5}{2}\hbar\omega$, $\frac{7}{2}\hbar\omega$, $\cdots$, which corresponds to $n_x+n_y+n_z = 0$, $n_x+n_y+n_z = 1$, $n_x+n_y+n_z = 2$, $\cdots$, respectively. Therefore, the degeneracy d of the nth energy $\left(n+\frac{3}{2}\right)\hbar\omega$ is equal to the number of (n_x, n_y, n_z) satisfying $n_x+n_y+n_z = n-1$,

$$d = \binom{n+1}{2} = \frac{(n+1)n}{2}. \tag{6}$$

A tetrahedral cluster consists of the 1-atom layer, 3-atom layer, 6-atom layer, 10-atom layer, $\cdots$. Suppose that a layer with fewer atoms is labeled by a smaller number. The number of atoms in the nth layer is given by

$$\binom{n+1}{2} = \frac{(n+1)n}{2}, \tag{7}$$

because it is equal to the number of atomic positions (i, j, k) satisfying $i+j+k = n-1$ (Supplementary Fig. 13). Because of the one-to-one correspondence $(i, j, k) \leftrightarrow (n_x, n_y, n_z)$, the number of atoms in the nth layer is equal to the degeneracy of the nth energy of the 3-dimensional isotropic harmonic oscillator. Therefore, if each atom has 2 valence electrons, the total number of valence electrons coincides with a magic number for the closed shell. The total number of atoms in a tetrahedral cluster with n layers is easily obtained as

$$\sum_{n'=1}^{n} \frac{(n'+1)n'}{2} = \frac{(n+2)(n+1)n}{3\cdot 2\cdot 1} = \binom{n+2}{3}. \tag{8}$$

Supplementary Figures

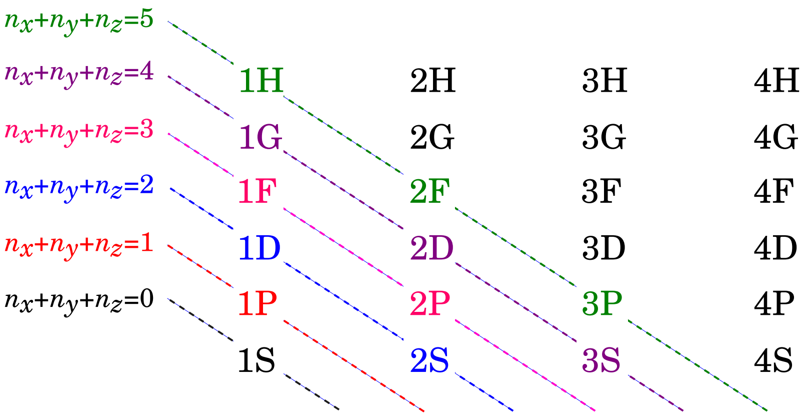

Supplementary Figure 1: Subduction of eigenstates of the 3-dimensional isotropic harmonic oscillator of $U(3)$ into those of the jellium model of $O(3)$.

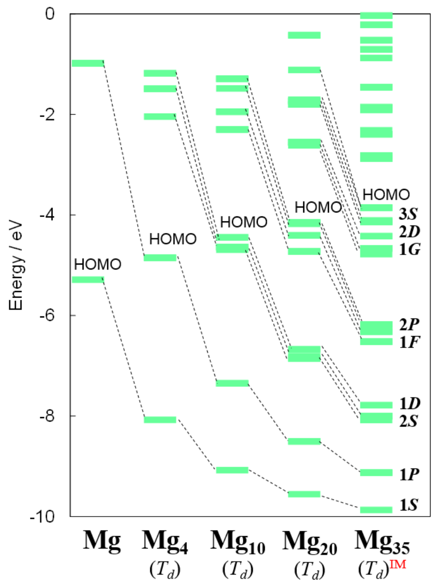

Supplementary Figure 2: Molecular orbital levels of Mg_1, Mg_4, Mg_{10}, Mg_{20} and Mg_{35} with the B3LYP/LanL2DZ level of theory. It should be noted that only Mg_{35} favors a lower symmetry due to the pseudo Jahn–Teller effect but was constrained to have the T_d symmetry for comparison.

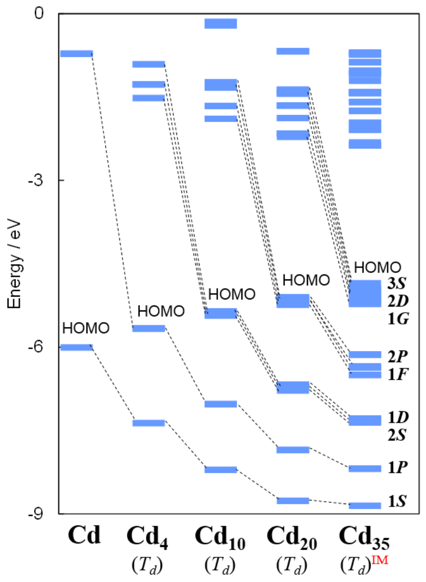

Supplementary Figure 3: Molecular orbital levels of Cd_1, Cd_4, Cd_{10} and Cd_{20} with the B3LYP/LanL2DZ level of theory. It should be noted that only Cd_{35} favors a lower symmetry due to the pseudo Jahn–Teller effect but was constrained to have the T_d symmetry for comparison.

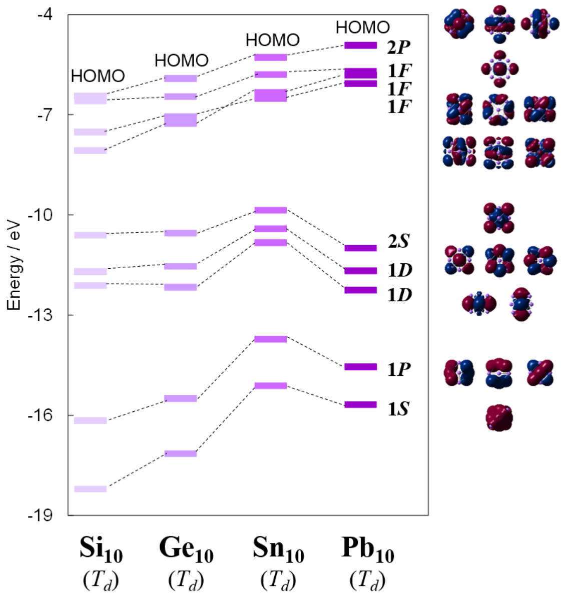

Supplementary Figure 4: Molecular orbital levels of Si_{10}, Ge_{10}, Sn_{10} and Pb_{10} with the B3LYP/LanL2DZ level of theory.

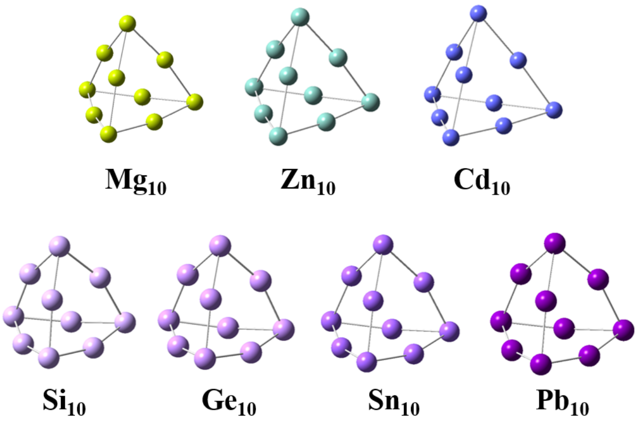

Supplementary Figure 5: Optimized geometries of Mg_{10}, Zn_{10}, Cd_{10}, Si_{10}, Ge_{10}, Sn_{10} and Pb_{10} with the B3LYP/LanL2DZ level of theory.

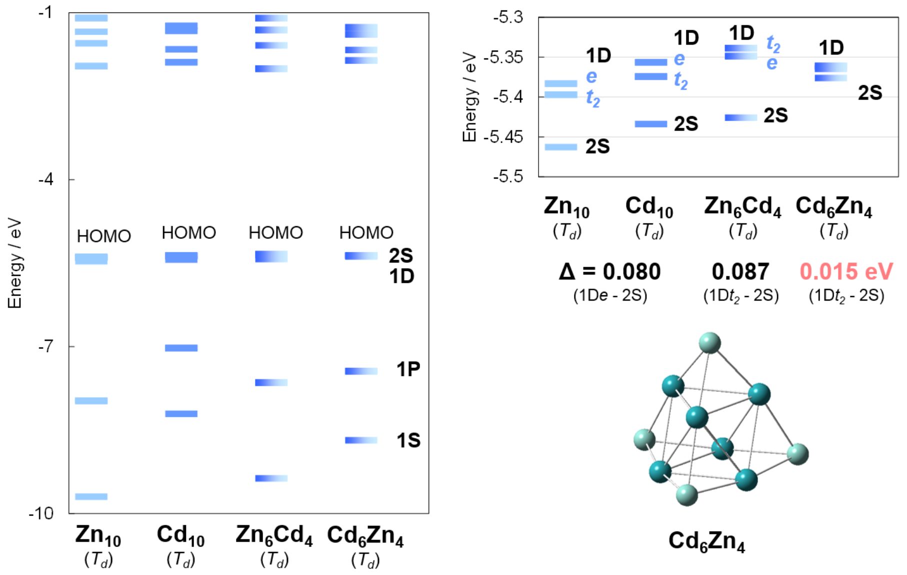

Supplementary Figure 6: Molecular orbital levels of Zn_{10}, Zn_{10}, Zn_6Cd_4 and Cd_6Zn_4 with the B3LYP/LanL2DZ level of theory.

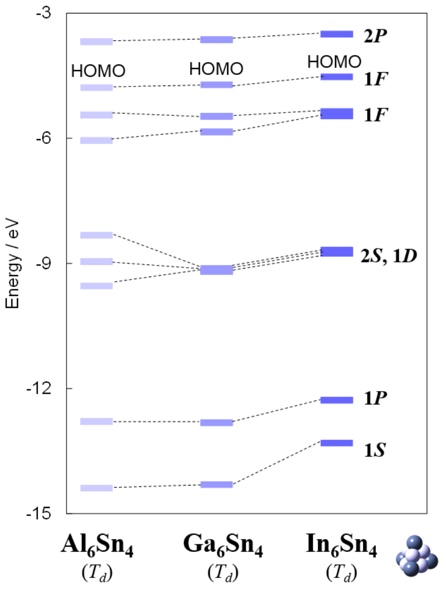

Supplementary Figure 7: Molecular orbital levels of Al_6Sn_4, Ga_6Sn_4 and In_6Sn_4 with the B3LYP/LanL2DZ level of theory.

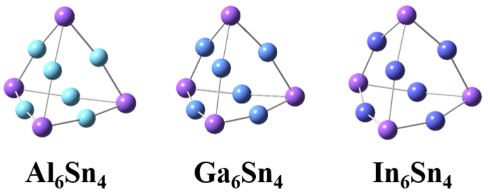

Supplementary Figure 8: Optimized geometries of Al_6Sn_4, Ga_6Sn_4 and In_6Sn_4 with the B3LYP/LanL2DZ level of theory.

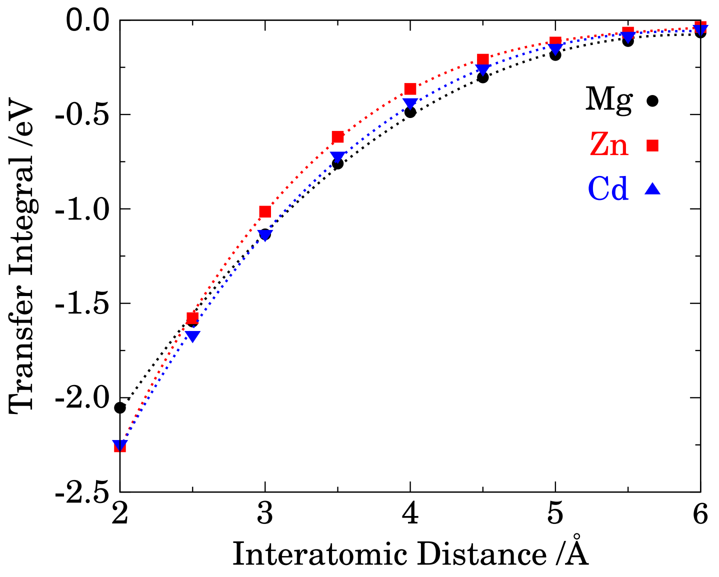

Supplementary Figure 9: Transfer integrals estimated as half the level of splitting between bonding and anti-bonding orbitals of *s*-type valence electrons with the B3LYP/LanL2DZ level of theory.

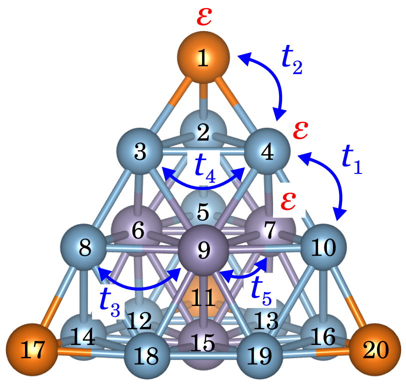

Supplementary Figure 10: Model parameters for the 20 atom system.

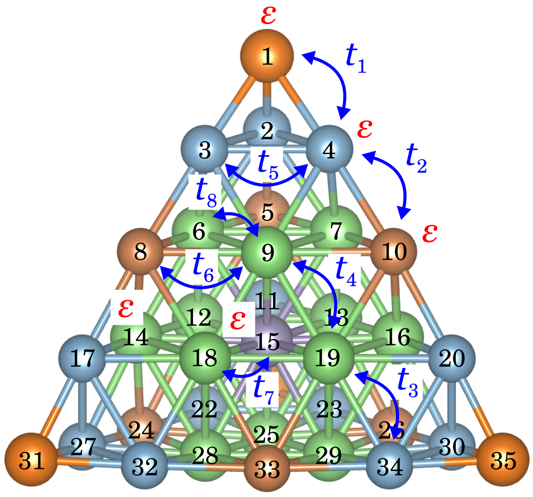

Supplementary Figure 11: Model parameters for the 35 atom system.

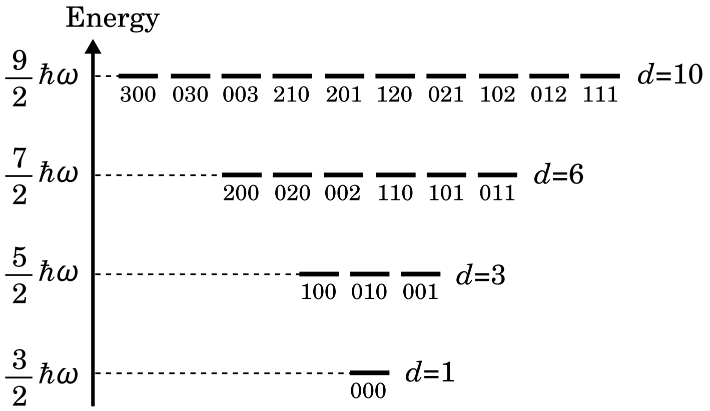

Supplementary Figure 12: Energy levels of the 3-dimensional isotropic harmonic oscillator.

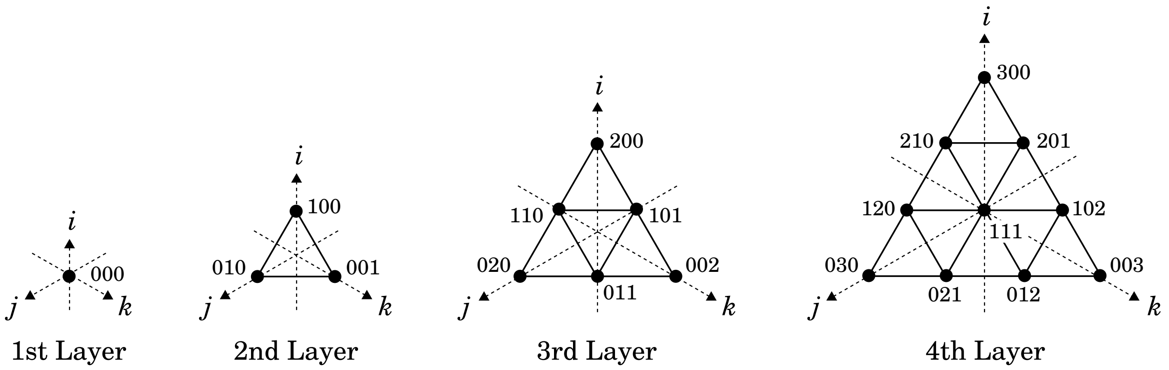

Supplementary Figure 13: The labels of atomic positions in each layer of a tetrahedral cluster.

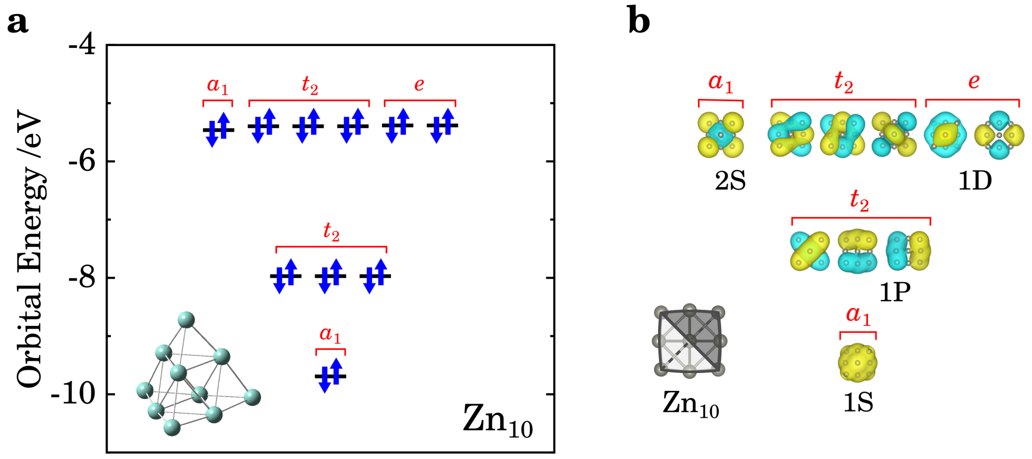

Supplementary Figure 14: **a** Occupied molecular orbital levels of Zn_{10} with their irreducible representations and the optimized geometry at the B3LYP/LanL2DZ level of theory. **b** Molecular orbitals of Zn_{10}, the isosurface values of which are 1.0×10^{-2} a.u.

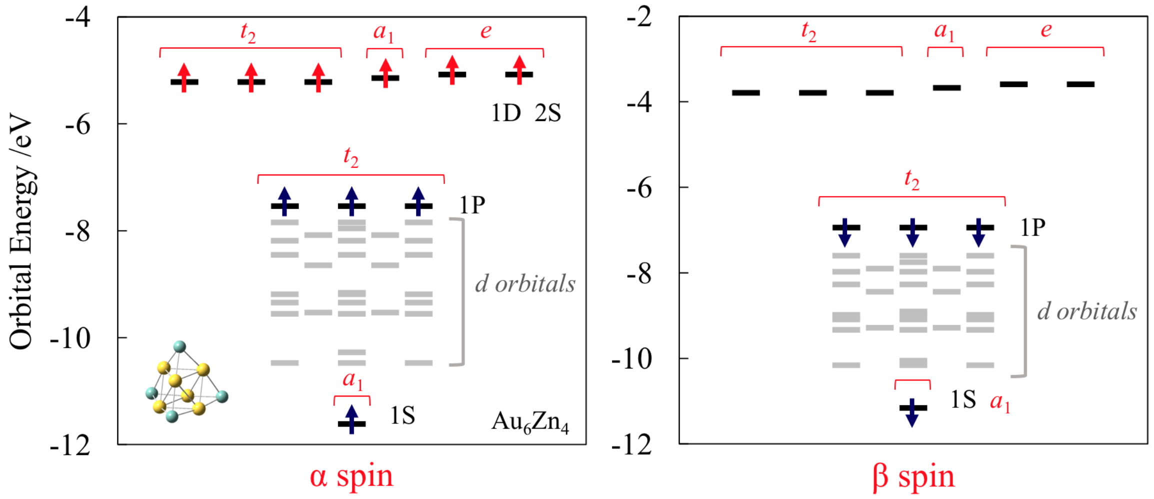

Supplementary Figure 15: Molecular orbital levels of Tl_{10} for α and β spins with its 11-et spin state at the UB3LYP/LanL2DZ level of theory. The Au and Zn elements have 1 and 2 valence electrons, respectively. Therefore, Au_6Zn_4 has a total of 14 valence electrons, producing the $(1S)^2$ $(1P)^6$ $(1D,2S)^6$ electronic configuration.

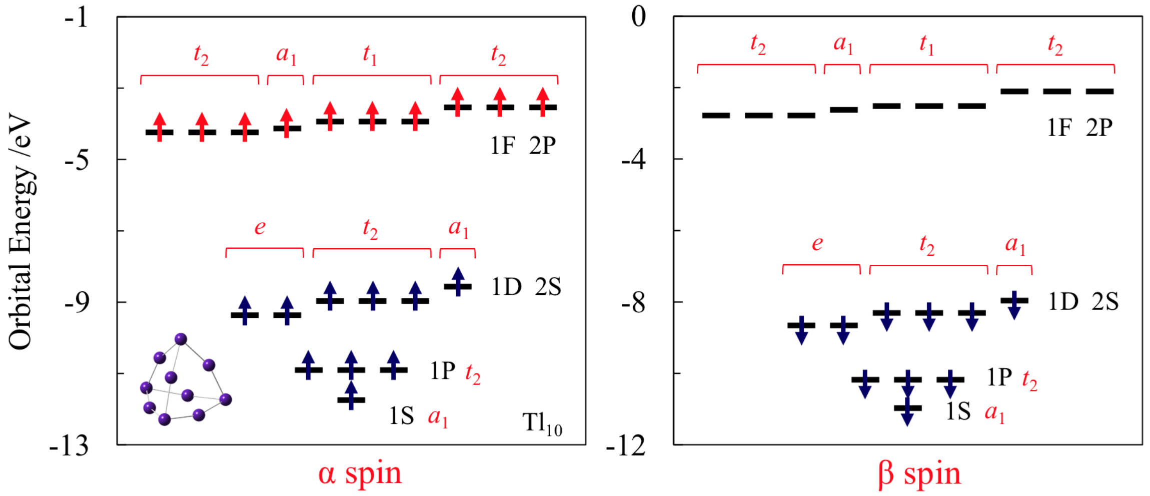

Supplementary Figure 16: Molecular orbital levels of Tl_{10} for α and β spins with its 11-et spin state at the UB3LYP/LanL2DZ level of theory. The Tl element has 3 valence electrons. Therefore, Tl_{10} has a total of 30 valence electrons, producing the $(1S)^2$ $(1P)^6$ $(1D,2S)^{12}$ $(1F,2P)^{10}$ electronic configuration.

Supplementary Tables

Supplementary Table 1: The optimized geometry of Mg_4 at the B3LYP/LanL2DZ level of theory.

Atom	Cartesian Coordinates /Å		
	x	*y*	*z*
Mg	1.109564	1.109564	1.109564
Mg	-1.109564	-1.109564	1.109564
Mg	1.109564	-1.109564	-1.109564
Mg	-1.109564	1.109564	-1.109564

Supplementary Table 2: The optimized geometry of Mg_{10} at the B3LYP/LanL2DZ level of theory.

Atom	Cartesian Coordinates /Å		
	x	*y*	*z*
Mg	-2.124221	2.124221	2.124221
Mg	0.000000	0.000000	2.380937
Mg	2.124221	-2.124221	2.124221
Mg	0.000000	2.380937	0.000000
Mg	0.000000	-2.380937	0.000000
Mg	-2.380937	0.000000	0.000000
Mg	-2.124221	-2.124221	-2.124221
Mg	0.000000	0.000000	-2.380937
Mg	2.124221	2.124221	-2.124221
Mg	2.380937	0.000000	0.000000

Supplementary Table 3: The optimized geometry of Mg_{20} at the B3LYP/LanL2DZ level of theory.

Atom	Cartesian Coordinates /Å		
	x	*y*	*z*
Mg	3.138073	3.138073	3.138073
Mg	1.023116	3.429500	1.023116
Mg	3.429500	1.023116	1.023116
Mg	1.023116	1.023116	3.429500
Mg	-1.023116	3.429500	-1.023116
Mg	-1.346094	1.346094	1.346094
Mg	-1.023116	-1.023116	3.429500
Mg	1.346094	-1.346094	1.346094
Mg	1.346094	1.346094	-1.346094
Mg	3.429500	-1.023116	-1.023116
Mg	-1.023116	1.023116	-3.429500
Mg	1.023116	-3.429500	-1.023116
Mg	1.023116	-1.023116	-3.429500
Mg	3.138073	-3.138073	-3.138073
Mg	-1.346094	-1.346094	-1.346094
Mg	-3.429500	1.023116	-1.023116
Mg	-1.023116	-3.429500	1.023116
Mg	-3.429500	-1.023116	1.023116
Mg	-3.138073	-3.138073	3.138073
Mg	-3.138073	3.138073	-3.138073

Supplementary Table 4: The optimized geometry of Mg_{35} at the B3LYP/LanL2DZ level of theory. It should be noted that it favors a lower symmetry due to the pseudo Jahn–Teller effect but was constrained to have the T_d symmetry for comparison.

Atom	Cartesian Coordinates /Å		
	x	y	z
Mg	4.163008	4.163008	4.163008
Mg	2.034480	4.454722	2.034480
Mg	2.034480	2.034480	4.454722
Mg	4.454722	2.034480	2.034480
Mg	-0.264108	2.401404	2.401404
Mg	-0.000000	4.642119	0.000000
Mg	-2.401404	2.401404	0.264108
Mg	2.401404	2.401404	-0.264108
Mg	-4.642119	0.000000	-0.000000
Mg	-2.401404	0.264108	2.401404
Mg	2.401404	-0.264108	2.401404
Mg	0.000000	0.000000	4.642119
Mg	-2.034480	-2.034480	4.454722
Mg	-4.163008	-4.163008	4.163008
Mg	0.264108	-2.401404	2.401404
Mg	-2.034480	-4.454722	2.034480
Mg	2.401404	-2.401404	0.264108
Mg	-4.454722	-2.034480	2.034480
Mg	4.642119	-0.000000	0.000000
Mg	0.000000	0.000000	0.000000
Mg	-2.401404	-2.401404	-0.264108

Mg	0.000000	-4.642119	0.000000
Mg	4.454722	-2.034480	-2.034480
Mg	2.034480	-4.454722	-2.034480
Mg	4.163008	-4.163008	-4.163008
Mg	2.034480	-2.034480	-4.454722
Mg	-0.264108	-2.401404	-2.401404
Mg	2.401404	0.264108	-2.401404
Mg	-2.401404	-0.264108	-2.401404
Mg	-0.000000	0.000000	-4.642119
Mg	0.264108	2.401404	-2.401404
Mg	-4.454722	2.034480	-2.034480
Mg	-2.034480	2.034480	-4.454722
Mg	-4.163008	4.163008	-4.163008
Mg	-2.034480	4.454722	-2.034480

Supplementary Table 5: The optimized geometry of Zn_4 at the B3LYP/LanL2DZ level of theory.

Atom	Cartesian Coordinates /Å		
	x	*y*	*z*
Zn	1.193203	1.193203	1.193203
Zn	-1.193203	-1.193203	1.193203
Zn	1.193203	-1.193203	-1.193203
Zn	-1.193203	1.193203	-1.193203

Supplementary Table 6: The optimized geometry of Zn_{10} at the B3LYP/LanL2DZ level of theory.

Atom	Cartesian Coordinates /Å		
	x	*y*	*z*
Zn	2.195452	2.195452	2.195452
Zn	0.000000	0.000000	2.368029
Zn	0.000000	2.368029	0.000000
Zn	-2.195452	-2.195452	2.195452
Zn	0.000000	-2.368029	0.000000
Zn	2.368029	0.000000	0.000000
Zn	2.195452	-2.195452	-2.195452
Zn	0.000000	0.000000	-2.368029
Zn	-2.368029	0.000000	0.000000
Zn	-2.195452	2.195452	-2.195452

Supplementary Table 7: The optimized geometry of Zn_{20} at the B3LYP/LanL2DZ level of theory.

Atom	Cartesian Coordinates /Å		
	x	*y*	*z*
Zn	-3.172017	3.172017	3.172017
Zn	-1.039938	3.475246	1.039938
Zn	1.039938	3.475246	-1.039938
Zn	3.172017	3.172017	-3.172017
Zn	3.475246	1.039938	-1.039938
Zn	1.204539	1.204539	1.204539
Zn	-1.039938	1.039938	3.475246
Zn	-3.475246	1.039938	1.039938
Zn	-3.475246	-1.039938	-1.039938
Zn	-1.204539	-1.204539	1.204539
Zn	1.039938	-1.039938	3.475246
Zn	3.475246	-1.039938	1.039938
Zn	3.172017	-3.172017	3.172017
Zn	1.039938	-3.475246	1.039938
Zn	-1.039938	-3.475246	-1.039938
Zn	-3.172017	-3.172017	-3.172017
Zn	-1.039938	-1.039938	-3.475246
Zn	-1.204539	1.204539	-1.204539
Zn	1.039938	1.039938	-3.475246
Zn	1.204539	-1.204539	-1.204539

Supplementary Table 8: The optimized geometry of Zn_{35} at the B3LYP/LanL2DZ level of theory. It should be noted that it favors a lower symmetry due to the pseudo Jahn–Teller effect but was constrained to have the T_d symmetry for comparison.

Atom	Cartesian Coordinates /Å		
	x	*y*	*z*
Zn	4.045569	4.045569	4.045569
Zn	1.996647	1.996647	4.573105
Zn	0.000000	0.000000	4.574283
Zn	-1.996647	-1.996647	4.573105
Zn	-4.045569	-4.045569	4.045569
Zn	-4.573105	-1.996647	1.996647
Zn	-2.289087	0.138496	2.289087
Zn	-0.138496	2.289087	2.289087
Zn	1.996647	4.573105	1.996647
Zn	4.573105	1.996647	1.996647
Zn	0.000000	4.574283	-0.000000
Zn	2.289087	-0.138496	2.289087
Zn	-1.996647	4.573105	-1.996647
Zn	-2.289087	2.289087	0.138496
Zn	0.138496	-2.289087	2.289087
Zn	-4.573105	1.996647	-1.996647
Zn	-4.574283	0.000000	-0.000000
Zn	-1.996647	-4.573105	1.996647
Zn	-2.289087	-2.289087	-0.138496
Zn	-4.045569	4.045569	-4.045569
Zn	0.000000	0.000000	0.000000

Zn	2.289087	2.289087	-0.138496
Zn	4.574283	-0.000000	0.000000
Zn	0.138496	2.289087	-2.289087
Zn	-2.289087	-0.138496	-2.289087
Zn	-0.000000	-4.574283	0.000000
Zn	-1.996647	1.996647	-4.573105
Zn	2.289087	-2.289087	0.138496
Zn	2.289087	0.138496	-2.289087
Zn	0.000000	-0.000000	-4.574283
Zn	-0.138496	-2.289087	-2.289087
Zn	1.996647	-4.573105	-1.996647
Zn	1.996647	-1.996647	-4.573105
Zn	4.045569	-4.045569	-4.045569
Zn	4.573105	-1.996647	-1.996647

Supplementary Table 9: The optimized geometry of Cd_4 at the B3LYP/LanL2DZ level of theory.

Atom	Cartesian Coordinates /Å		
	x	*y*	*z*
Cd	1.389113	1.389113	1.389113
Cd	-1.389113	-1.389113	1.389113
Cd	1.389113	-1.389113	-1.389113
Cd	-1.389113	1.389113	-1.389113

Supplementary Table 10: The optimized geometry of Cd_{10} at the B3LYP/LanL2DZ level of theory.

Atom	Cartesian Coordinates /Å		
	x	*y*	*z*
Cd	2.655376	2.655376	-2.655376
Cd	0.000000	2.798783	0.000000
Cd	0.000000	0.000000	-2.798783
Cd	-2.655376	-2.655376	-2.655376
Cd	-2.798783	0.000000	0.000000
Cd	0.000000	-2.798783	0.000000
Cd	0.000000	0.000000	2.798783
Cd	-2.655376	2.655376	2.655376
Cd	2.655376	-2.655376	2.655376
Cd	2.798783	0.000000	0.000000

Supplementary Table 11: The optimized geometry of Cd_{20} at the B3LYP/LanL2DZ level of theory.

Atom	Cartesian Coordinates /Å		
	x	*y*	*z*
Cd	3.888158	3.888158	-3.888158
Cd	1.283883	4.110424	-1.283883
Cd	1.283883	1.283883	-4.110424
Cd	-1.283883	-1.283883	-4.110424
Cd	-3.888158	-3.888158	-3.888158
Cd	-1.283883	-4.110424	-1.283883
Cd	-4.110424	-1.283883	-1.283883
Cd	1.403285	-1.403285	-1.403285
Cd	-1.403285	1.403285	-1.403285
Cd	4.110424	1.283883	-1.283883
Cd	-1.283883	4.110424	1.283883
Cd	-3.888158	3.888158	3.888158
Cd	-1.283883	1.283883	4.110424
Cd	1.403285	1.403285	1.403285
Cd	-4.110424	1.283883	1.283883
Cd	4.110424	-1.283883	1.283883
Cd	-1.403285	-1.403285	1.403285
Cd	1.283883	-4.110424	1.283883
Cd	3.888158	-3.888158	3.888158
Cd	1.283883	-1.283883	4.110424

Supplementary Table 12: The optimized geometry of Cd_{35} at the B3LYP/LanL2DZ level of theory. It should be noted that it favors a lower symmetry due to the pseudo Jahn–Teller effect but was constrained to have the T_d symmetry for comparison.

Atom	Cartesian Coordinates /Å		
	x	y	z
Cd	-5.317444	5.317444	5.317444
Cd	-2.635633	5.634596	2.635633
Cd	-5.634596	2.635633	2.635633
Cd	-2.635633	2.635633	5.634596
Cd	0.000000	5.464731	0.000000
Cd	-2.807883	2.807883	0.003613
Cd	-0.003613	2.807883	2.807883
Cd	-2.807883	0.003613	2.807883
Cd	2.635633	5.634596	-2.635633
Cd	-5.464731	0.000000	0.000000
Cd	0.003613	2.807883	-2.807883
Cd	0.000000	0.000000	0.000000
Cd	2.807883	2.807883	-0.003613
Cd	-0.000000	0.000000	5.464731
Cd	2.807883	-0.003613	2.807883
Cd	2.635633	-2.635633	5.634596
Cd	0.003613	-2.807883	2.807883
Cd	5.634596	2.635633	-2.635633
Cd	2.807883	-2.807883	0.003613
Cd	5.464731	-0.000000	0.000000
Cd	2.635633	-5.634596	2.635633

Cd	5.634596	-2.635633	2.635633
Cd	5.317444	-5.317444	5.317444
Cd	0.000000	-5.464731	0.000000
Cd	2.807883	0.003613	-2.807883
Cd	5.317444	5.317444	-5.317444
Cd	-2.807883	-2.807883	-0.003613
Cd	-2.807883	-0.003613	-2.807883
Cd	-5.634596	-2.635633	-2.635633
Cd	2.635633	2.635633	-5.634596
Cd	-0.000000	-0.000000	-5.464731
Cd	-2.635633	-2.635633	-5.634596
Cd	-5.317444	-5.317444	-5.317444
Cd	-2.635633	-5.634596	-2.635633
Cd	-0.003613	-2.807883	-2.807883

Supplementary Table 13: The optimized geometry of Si_{10} at the B3LYP/LanL2DZ level of theory.

Atom	Cartesian Coordinates /Å		
	x	*y*	*z*
Si	1.693816	1.693816	-1.693816
Si	0.000000	2.127154	0.000000
Si	2.127154	0.000000	0.000000
Si	0.000000	0.000000	-2.127154
Si	-1.693816	-1.693816	-1.693816
Si	0.000000	-2.127154	0.000000
Si	1.693816	-1.693816	1.693816
Si	-2.127154	0.000000	0.000000
Si	0.000000	0.000000	2.127154
Si	-1.693816	1.693816	1.693816

Supplementary Table 14: The optimized geometry of Ge_{10} at the B3LYP/LanL2DZ level of theory.

Atom	Cartesian Coordinates /Å		
	x	*y*	*z*
Ge	-1.794420	1.794420	1.794420
Ge	0.000000	2.276197	0.000000
Ge	0.000000	0.000000	2.276197
Ge	-2.276197	0.000000	0.000000
Ge	1.794420	-1.794420	1.794420
Ge	0.000000	0.000000	-2.276197
Ge	2.276197	0.000000	0.000000
Ge	1.794420	1.794420	-1.794420
Ge	0.000000	-2.276197	0.000000
Ge	-1.794420	-1.794420	-1.794420

Supplementary Table 15: The optimized geometry of Sn_{10} at the B3LYP/LanL2DZ level of theory.

Atom	Cartesian Coordinates /Å		
	x	*y*	*z*
Sn	2.047865	2.047865	2.047865
Sn	0.000000	0.000000	2.504962
Sn	-2.047865	-2.047865	2.047865
Sn	0.000000	2.504962	0.000000
Sn	-2.504962	0.000000	0.000000
Sn	-2.047865	2.047865	-2.047865
Sn	0.000000	0.000000	-2.504962
Sn	0.000000	-2.504962	0.000000
Sn	2.504962	0.000000	0.000000
Sn	2.047865	-2.047865	-2.047865

Supplementary Table 16: The optimized geometry of Pb_{10} at the B3LYP/LanL2DZ level of theory.

Atom	Cartesian Coordinates /Å		
	x	*y*	*z*
Pb	-2.080380	2.080380	2.080380
Pb	0.000000	2.626407	0.000000
Pb	-2.626407	0.000000	0.000000
Pb	0.000000	-2.626407	0.000000
Pb	0.000000	0.000000	2.626407
Pb	2.080380	-2.080380	2.080380
Pb	-2.080380	-2.080380	-2.080380
Pb	0.000000	0.000000	-2.626407
Pb	2.080380	2.080380	-2.080380
Pb	2.626407	0.000000	0.000000

Supplementary Table 17: The optimized geometry of Zn_6Cd_4 at the B3LYP/LanL2DZ level of theory.

Atom	Cartesian Coordinates /Å		
	x	*y*	*z*
Zn	0.000000	2.407245	0.000000
Zn	-2.407245	0.000000	0.000000
Zn	2.407245	-0.000000	0.000000
Zn	-0.000000	-0.000000	-2.407245
Zn	0.000000	-2.407245	0.000000
Zn	-0.000000	0.000000	2.407245
Cd	-2.352431	-2.352431	2.352431
Cd	2.352431	2.352431	2.352431
Cd	-2.352431	2.352431	-2.352431
Cd	2.352431	-2.352431	-2.352431

Supplementary Table 18: The optimized geometry of Cd_6Zn_4 at the B3LYP/LanL2DZ level of theory.

Atom	Cartesian Coordinates /Å		
	x	*y*	*z*
Cd	0.000000	0.000000	2.710524
Cd	0.000000	2.710524	-0.000000
Cd	-2.710524	0.000000	-0.000000
Cd	0.000000	-0.000000	-2.710524
Cd	-0.000000	-2.710524	0.000000
Cd	2.710524	-0.000000	0.000000
Zn	2.411128	2.411128	2.411128
Zn	-2.411128	-2.411128	2.411128
Zn	2.411128	-2.411128	-2.411128
Zn	-2.411128	2.411128	-2.411128

Supplementary Table 19: The optimized geometry of Al_6Sn_4 at the B3LYP/LanL2DZ level of theory.

Atom	Cartesian Coordinates /Å		
	x	*y*	*z*
Sn	-1.994720	1.994720	1.994720
Sn	1.994720	-1.994720	1.994720
Sn	-1.994720	-1.994720	-1.994720
Sn	1.994720	1.994720	-1.994720
Al	0.000000	0.000000	2.193171
Al	0.000000	2.193171	0.000000
Al	-2.193171	0.000000	0.000000
Al	0.000000	-2.193171	0.000000
Al	0.000000	0.000000	-2.193171
Al	2.193171	0.000000	0.000000

Supplementary Table 20: The optimized geometry of Ga_6Sn_4 at the B3LYP/LanL2DZ level of theory.

Atom	Cartesian Coordinates /Å		
	x	*y*	*z*
Sn	1.983154	1.983154	1.983154
Sn	-1.983154	-1.983154	1.983154
Sn	-1.983154	1.983154	-1.983154
Sn	1.983154	-1.983154	-1.983154
Ga	0.000000	2.290672	0.000000
Ga	2.290672	0.000000	0.000000
Ga	0.000000	0.000000	2.290672
Ga	0.000000	-2.290672	0.000000
Ga	-2.290672	0.000000	0.000000
Ga	0.000000	0.000000	-2.290672

Supplementary Table 21: The optimized geometry of In_6Sn_4 at the B3LYP/LanL2DZ level of theory.

Atom	Cartesian Coordinates /Å		
	x	*y*	*z*
Sn	2.080353	2.080353	2.080353
Sn	-2.080353	-2.080353	2.080353
Sn	-2.080353	2.080353	-2.080353
Sn	2.080353	-2.080353	-2.080353
In	0.000000	2.572321	0.000000
In	2.572321	0.000000	0.000000
In	0.000000	0.000000	2.572321
In	0.000000	-2.572321	0.000000
In	0.000000	0.000000	-2.572321
In	-2.572321	0.000000	0.000000

Supplementary Table 22: The optimized geometry of Au_6Zn_4 with its septet spin state at the B3LYP/LanL2DZ level of theory. This is one of stable states with no imaginary frequency.

Atom	Cartesian Coordinates /Å		
	x	*y*	*z*
Au	0.000000	2.159438	0.000000
Au	2.159438	-0.000000	0.000000
Au	-0.000000	0.000000	2.159438
Au	-0.000000	-2.159438	0.000000
Au	-2.159438	0.000000	0.000000
Au	-0.000000	0.000000	-2.159438
Zn	1.936069	-1.936069	-1.936069
Zn	-1.936069	1.936069	-1.936069
Zn	1.936069	1.936069	1.936069
Zn	-1.936069	-1.936069	1.936069

Supplementary Table 23: The optimized geometry of Tl_{10} with its 11-et spin state at the B3LYP/LanL2DZ level of theory. This is one of stable states with no imaginary frequency.

Atom	Cartesian Coordinates /Å		
	x	*y*	*z*
Tl	-0.000000	2.961903	0.000000
Tl	-0.000000	0.000000	2.961903
Tl	-2.961903	0.000000	0.000000
Tl	0.000000	-2.961903	0.000000
Tl	-0.000000	-0.000000	-2.961903
Tl	2.961903	-0.000000	0.000000
Tl	2.384484	-2.384484	2.384484
Tl	-2.384484	2.384484	2.384484
Tl	-2.384484	-2.384484	-2.384484
Tl	2.384484	2.384484	-2.384484
